# The Interaction of Osmotic and Heavy Metal Stress in C. elegans

**DOI:** 10.1093/geroni/igab046.2566

**Published:** 2021-12-17

**Authors:** Emily Turner, Amanda Furtmann, Hope Dang, Destiny DeNicola, George Sutphin

**Affiliations:** 1 University of Arizona, Tucson, Arizona, United States; 2 The University of Arizona, Tucson, Arizona, United States

## Abstract

Cellular stress is an ever-present aspect of aging and a primary driver of many common age-associated diseases such as cancer, diabetes, or neurodegenerative diseases. As we age, stress-induced damage accumulates over time, along with reduced efficacy of stress response pathways at combatting such damage. Molecular stress response pathways are well studied in the context of individual stressors, but there is a lack of understanding of how these responses change when multiple stressors are encountered at the same time. The goal of our work is to explore the impact of multiple simultaneous stressors on health and survival, and to investigate the underlying molecular pathways involved. To accomplish this, we utilize the nematode Caenorhabditis elegans to monitor lifespan changes in response to various stressors. We simultaneously exposed C. elegans to high concentrations of sodium chloride and cadmium chloride, known to induce osmotic and heavy metal stress, respectively. We found that lifespan is drastically decreased by the combined stress, significantly more so than the reduction in lifespan caused by either individual stress. Our results show that glycerol levels, which are normally increased in response to osmotic stress, are significantly lowered when the two stresses are combined compared to levels detected for osmotic stress alone. This suggests that the presence of cadmium may sensitize worms to sodium and other osmotic stressors by blunting cells’ ability to mount an appropriate molecular response. In ongoing work, we will continue to dissect the mechanisms through which cadmium influences glycerol production and other aspects of osmotic stress response.

